# Discovering irregular pupil light responses to chromatic stimuli using waveform shapes of pupillograms

**DOI:** 10.1186/s13637-014-0018-x

**Published:** 2014-10-07

**Authors:** Minoru Nakayama, Wioletta Nowak, Hitoshi Ishikawa, Ken Asakawa, Yoshiaki Ichibe

**Affiliations:** 1 Department of Human System Science, Tokyo Institute of Technology, Ookayama, Meguro, Tokyo, 152-8552 Japan; 2 Institute of Biomedical Engineering and Instrumentation, Wroclaw University of Technology, Wroclaw, 50-370 Poland; 3 School of Allied Health Sciences, Kitasato University, Kitasato, Minami, Sagamihara, 252-0373 Japan; 4 School of Medicine, Kitasato University, Kitasato, Minami, Sagamihara, 252-0373 Japan

## Abstract

**Background:**

The waveforms of the pupillary light reflex (PLR) can be analyzed in a diagnostic test that allows for differentiation between disorders affecting photoreceptors and disorders affecting retinal ganglion cells, using various signal processing techniques. This procedure has been used on both healthy subjects and patients with age-related macular degeneration (AMD), as a simple diagnostic procedure is required for diagnosis.

**Results:**

The Fourier descriptor technique is used to extract the features of PLR waveform shapes of pupillograms and their amplitudes. To detect those patients affected by AMD using the extracted features, multidimensional scaling (MDS) and clustering techniques were used to emphasize stimuli and subject differences. The detection performance of AMD using the features and the MDS technique shows only a qualitative tendency, however. To evaluate the detection performance quantitatively, a set of combined features was created to evaluate characteristics of the PLR waveform shapes in detail. Classification performance was compared across three categories (AMD patients, aged, and healthy subjects) using the Random Forest method, and weighted values were optimized using variations of the classification error rates. The results show that the error rates for healthy pupils and AMD-affected pupils were low when the value of the coefficient for a combination of PLR amplitudes and features of waveforms was optimized as 1.5. However, the error rates for patients with age-affected eyes was not low.

**Conclusions:**

A classification procedure for AMD patients has been developed using the features of PLR waveform shapes and their amplitudes. The results show that the error rates for healthy PLRs and AMD PLRs were low when the Random Forest method was used to produce the classification. The classification of pupils of patients with age-affected eyes should be carefully considered in order to produce optimum results.

**Electronic supplementary material:**

The online version of this article (doi:10.1186/s13637-014-0018-x) contains supplementary material, which is available to authorized users.

## 1Introduction

The pupillary response has long been used for diagnostic procedures [1] and psycho-physiological studies [2]-[4]. The pupillary response, a reaction to light intensity, is well-known, and the pupillary light response controls the dilation and constriction of the pupil in response to changes in light intensity. The response is usually observed and recorded using pupillograms which consist of pupil diameter and time diagrams [5].

This response is often called the pupillary light reflex (PLR). It has also been used as an objective measure of retinal and optic nerve functions. In recent years, a growth in interest in the examination of PLR has been observed. It is the result of the discovery of a new type of retinal ganglion cells [6],[7]. Since the melanopsin-associated photoreceptive system (ipRGCs, intrinsically photosensitive retinal ganglion cells) in the human retina was discovered [8], various diagnostic procedures have been proposed and introduced.

In particular, the difference between the response behavior of this photoreceptive system to some types of chromatic stimuli and the behavior of the conventional rod-cone system has been compared [8] to determine the possibility of detecting the condition of the retina. In addition to receiving rod and cone inputs, the responses based on the newly discovered system have been studied as intrinsically photosensitive [9],[10]. Those cells are interchangeably referred to as ipRGC or melanopsin-mediated retinal ganglion cells (mRGC), and according to recent studies, they drive pupillary responses and circadian rhythms [6]-[8]. Also, this phenomenon has been applied to diagnostic procedures for patients with glaucoma [11] or retinas pigmentosa [12],[13]. Feature extraction and analysis of PLRs has made a significant contribution to the development of diagnostic procedures for these patients, as opthalmologically scientific evidence was discovered. Another well-known disease which is observed in aging patients is age-related macular degeneration (AMD) [14]. This disease is related to the condition of the retina, and thus, PLRs are affected by the progress of the disease. Unfortunately, this disease spreads gradually. Therefore, detection and prediction procedures are required. A simple procedure which detects prominent symptoms, such as an easy at home test, is necessary.

In addition to some types of portable pupillometers currently available, a PC web camera and a smart phone with an additional lens can be used to observe pupillography. Some types of clinical consultations can be conducted anywhere using either of these pieces of equipment.

As mentioned above, PLRs can be applied to extract features and to detect irregular responses. This means that a record of temporal pupillary changes as pupillograms is a time series signal, requiring various types of signal processing techniques. This suggests that a simple signal processing technique may be used to extract some symptoms of the disease. For example, the Fourier descriptor technique is often used to indicate the shapes of waveforms [15]-[17], and the possibility of using this method should be examined.

In this paper, feature expressions of PLR waveform shapes of pupillograms are introduced to identify pupil characteristics. Also, the possibility of detecting eyes which are affected by AMD and other factors is examined. Therefore, the following topics are addressed:

1. A procedure for extracting features of PLR waveforms of pupillograms is created, and the features used to compare these waveforms are analyzed to detect irregular responses.
2. To determine the possibility of detection in diseased eyes, several classifying techniques were applied to features of PLRs, and the performance of the techniques is discussed.

## 2Related works

The study of ipRGC mentioned in related research work currently being conducted is concerned mainly with, on the one hand, the study of the ipRGC structure and functions and on the other hand, with the diagnostic use of their specific activity. A brief review of literature regarding this research is presented below.

The ipRGCs are atypical retinal photoreceptors distinct from classical rod and cone photoreceptors [18]. They express the photopigment melanopsin and are intrinsically photosensitive, since they showed sluggish melanopsin-mediated responses. They can also act as conventional RGCs by receiving synaptic rod/cone input via bipolar cells. This integrated information is then transmitted to numerous discrete brain regions involved in both non-image and image-forming vision [19]. The ipRGCs ultimately modulate a multiplicity of behaviors including circadian photoentrainment, PLR, activity masking, sleep/arousal, anxiety, light aversion, and even make a significant contribution to visual functions. Recently, it has been discovered that ipRGCs consist of several subtypes that are morphologically and physiologically distinct, which contribute differentially to the abovementioned non-image and image-forming functions [19],[20]. Detailed study of the ipRGC’s different types and their behaviour with varying attributes of light are being still analyzed. In particular, intensive research involving the relationship between melanopsin activity and PLR reflex is now being conducted [21],[22]. These studies have direct applications in clinical conditions for the diagnosis of retinal degeneration and sleep disturbances under clinical conditions [22],[23].

The application of ipRGCs to the study of retinal degeneration is focused mainly on the use of pupil responses to chromatic light as a clinical marker, to allow differentiation between disorders affecting rod/cone photoreceptors (the outer retina) and those affecting retinal ganglion cells (the inner retina). As used above, the terms ‘inner retina’ and ‘outer retina’ are the consequence of the anatomical distribution of ipRGC photoreceptors (the inner nuclear layer) and rod/cone photoreceptors (the outer retinal layer) in the retina [24]. Where a disease affects multiple retinal layers, the pupillary light reflex could be a useful tool in determining the contributions of the inner and outer retina to the disease process.

Kankipati et al. focused on the post illumination pupil response (PIPR) analysis of glaucoma patients, and compared them with normal subjects [25],[26]. They used a 10-s light stimulus (retinal irradiance, 13 log quanta/ c*m*^2^/s; light wavelengths, 470 nm (blue), and 623 nm (red)) and recorded pupillary response for 50 s after light cessation. They found that normal subjects displayed a significant PIPR for blue light (but not for red light), which is consistent with the proposed melanopsin-mediated response. When glaucoma patients are compared to patients using age-matching controls, there was a significant decrease in melanopsin-mediated PIPR. They concluded that PIPR has the potential for use as a clinical tool for evaluating patients with glaucoma. Feigl et al. [11] also tested PIPR to analyze whether glaucoma alters the function of ipRGC. They use a 10-s light stimuli with 488 nm and 610 nm light wavelengths and retinal irradiance of 14.2 log photons c*m*^2^/s. They have found that patients with advanced glaucoma have a dysfunctional ipRGC-mediated PIPR. It has been confirmed that PIPR may be a clinical indicator of progressive changes in glaucoma.

Kardon et al. focused on the percentage of pupil contraction in transient and sustained pupil responses in patients with retinas pigmentosa [12],[13]. They used a stimulus paradigm using red and blue light as a continuous Ganzfeld stimulus which produced a 13-s stepwise increase in intensity over a 2 log-unit range (low (1 cd/*m*^2^), medium (10 cd/*m*^2^), and high (100 cd/*m*^2^)). They have found that pupil responses to red and blue light stimuli which are weighed to favor cone or rod input are significantly reduced in patients with retinas pigmentosa. Their preliminary results suggest that pupil response to a low-intensity blue light, to a high-intensity red light, and to a high-intensity blue light may be reasonable markers of rod activity, cone-driven responses, and direct, intrinsic activation of mRGC, respectively.

AMD is another concern regarding retinal disease. This disease causes impairment of both the inner and outer retinal layers, depending on the stage of the disease, and pupil chromatic response measurements may allow the monitoring of the progression of the disease or facilitate the determination of different stages of the disease [27]. In particular, the function of ipRGC concerns the PLR while AMD influences the waveforms of PLR [22].

Brozou et al. tested outer retinal contributions to pupil responses in patients with AMD [28]. The study showed that AMD significantly affects the pupil’s response to light stimulus (20 msec duration and 24.6 cd/*m*^2^ intensity), when compared to normal subjects. Feigl and Zele present a new experimental paradigm for the first time that allows the differentiation of inner and outer retinal contributions to the pupil response in AMD [22]. They used a 11.9-s duration 0.5 Hz sine wave stimulus with 464 and 635 nm light wavelengths and retinal irradiance of 15.1 log photon c*m*^−2^*s*^−1^. Additionally, they introduced a new metric called ‘a phase amplitude percentage’ (PAP) that reflects inner and outer interactions. PAP is determined from the average long-wavelength and short-wavelength peak-to-through phase amplitudes. PAP approaches zero for retinal irradiances below a certain melanopsin threshold, as PLR is driven by rods and cones. PAP is non-zero for retinal irradiances above a certain melanopsin threshold, as PLR is predominantly driven by cones with ipRGC contributions. They also showed that application of this paradigm to AMD patients can provide information that ipRGCs are altered. This result can be a very important step toward using PLR to determine the inner and outer retinal dysfunction of AMD patients. Future work will be focused on quantifying retinal inputs to the pupil response in order to determine the different stages of AMD or to monitor the disease’s progression.

Our work presents an attempt to use the pupil response to chromatic rectangular light pulses, which are routinely measured in clinical studies, as a simple and fast tool (indicator) for early detection of AMD. AMD (both the dry and wet forms) is the one of the most common irreversible causes of severe loss of vision. It usually affects older adults and results in a loss of vision because of damage to the central part of the retina, known as the macula. The gradual disappearance of the retina pigment epithelium (RPE) in the dry form results in patches of chorioretinal atrophy lacking any visual function. In the wet form, the damage is due to the escape of subretinal fluid/intraretinal fluid, blood, or destruction of photoreceptors and RPE by fibrous or fibrovascular tissue. Since AMD destroys photoreceptors which are initial receptors for PLR, it may reduce the PLR. The type or localization of the damaged photoreceptors may then be indicated using a pupil response to chromatic light. AMD is painless and, consequently, the lack of early warning signals in existing retinal pathology necessitates an indicator which will allow for earlier and easier detection (prediction) of this type of disorder. It is believed that the pupil reaction to chromatic light could be such an indicator.

## 3Methods

### 3.1 Subjects

Six healthy young subjects (20 to 21 years old) and six elderly AMD-affected patients (59 to 86 years old) participated. Each elderly patient had a diseased eye and a normal eye, according to a medical doctor.

Diseased eyes with choroidal neovascularization (CNV) in the macular region are often affected by new blood vessels, which bleed and form dense macular scars [14]. Also, CNV is a major cause of visual loss due to AMD [14].

Patient details are summarized in Table 1. Regarding fluorescein angiographic assessment as a medical diagnostic [14], the diseased eyes are classified into *predominantly classic CNV lesion* (the area of classic CNV occupies 50% or more of the entire lesion) and *occult lesion* (either fibrovascular pigment epithelial detachments or late leakage from an undetermined source [29]). As regards to the clinical consultation, there is no significant difference in the seriousness of disease between the two types. Table 1 shows patients with normal eyes that have sufficient visual acuity.

**Table 1:** **Patient information**

				Visual acuity	
	Age	Sex	Type of disease	D	N
Patient 1	82	Male	Predominantly	0.2	1.0
Patient 2	66	Male	Predominantly	0.04	1.0
Patient 3	74	Male	Predominantly	0.4	0.9
Patient 4	86	Male	Occult	0.6	1.0
Patient 5	59	Male	Occult	0.5	1.2
Patient 6	63	Male	Occult	0.2	1.0

Predominantly, predominantly classic CNV; Occult, occult without classic CNV; D, disease eye; N, normal eye.

This study followed the tenets of the Declaration of Helsinki regarding research involving human subjects; informed consent was obtained from all subjects. The study protocol was approved at the Kitasato University School of Medicine Institutional Ethics Committee. This test does not determine the risk, however.

### 3.2 Experimental procedure

Pupil responses were measured using a PLR observation procedure and without any procedures to stimulate mydriasis, to determine the level of functionality of the melanopsin-associated photoreceptors [9],[10].

Pupil responses were recorded using Iriscorder Dual equipment (Hamamatsu Photonics, Hamamatsu, Japan) at a sampling rate of 30 Hz. This equipment is designed for observing PLRs with ipRGCs activation in accordance with the measurements taken in previous studies [10],[12] and consists of a measurement controller and a pair of goggles which have an infrared camera and a LED light source for each eye of the goggles. Therefore, the stimulus light presentation was binocular, and the recordings were also binocular. In the experiment, a long wavelength (635 ± 5 nm) red light and a short wavelength (470 ± 7 nm) blue light were used at two different light intensities (10 and 100 cd/*m*^2^). The light stimulation conditions were adjusted in accordance with the method of Kawasaki and Kardon [10],[12] while the measurement validity was also confirmed [30],[31]. PLRs for light stimuli were simply recorded as single trial measure. The light pulses were projected within the housing of a pair of goggles. Subjects were asked to not blink for 20 s while their pupil diameter was recorded. The observation period consisted of a 10-s light pulse which caused a restriction of the pupil size, followed by 10 s without a light pulse during which restoration of the pupil size was allowed to occur. These measurements were taken in a dark room with constant lighting conditions. A dark adaptation period of 5 min was allowed prior to the taking of measurements.

**Figure 1:**
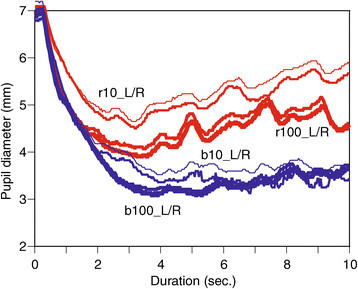
**Healthy subject reactions for both eyes.**

**Figure 2:**
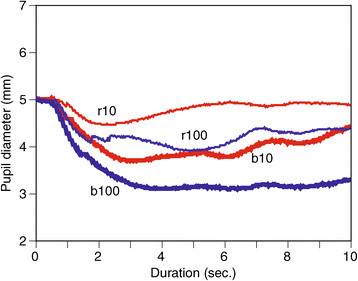
**Pupil light reflex on diseased eye of an AMD patient.**

Responses for both left and right pupils were recorded as pupillograms for each subject. In this paper, the four conditions for the left (L) and right (R) eyes are defined as follows: ‘r10’ (long wavelength and low light intensity), ‘r100’ (long and high intensity), ‘b10’ (short wavelength and low intensity), and ‘b100’ (short wavelength and high intensity). The pupil responses of a healthy subject are illustrated in Figure 1, and the pupil responses of a patient diseased eye with AMD are illustrated in Figure 2. In both figures, the red line indicates pupil responses for red light, and the blue line indicates responses for blue light. In Figure 1, the very fine line shows the pupil response of the left eye for 10 cd/*m*^2^, the less fine line shows the pupil response of the right eye for 10 cd/*m*^2^, while the bold line shows pupil response of left eye for 100 cd/*m*^2^ and the bolder line shows pupil response for the right eye for 100 cd/*m*^2^.

In Figure 1, there is little difference in PLR waveform shapes between the left and right eyes. Pupil sizes are sustained during both blue light pulses, and restoration of pupil sizes can be observed after both red light pulses. These phenomena confirm pupil behavior using blue and red light pulses observed in previous studies. However, these phenomena influenced diseased eyes, as shown in Figure 2.

**Figure 3:**
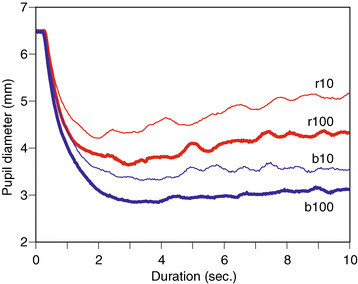
**Averaged pupil light reflex on healthy eyes.**

**Figure 4:**
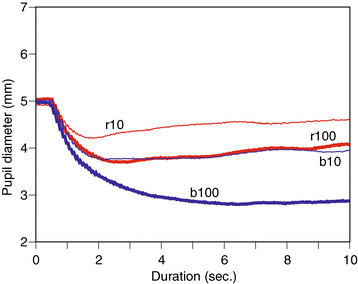
**Averaged pupil light reflex on diseased eyes.**

To emphasize the difference in PLR waveforms between patient’s diseased eye and healthy eyes, averaged PLRs are illustrated for all healthy eyes in Figure 3 and for all diseased eyes in Figure 4. Regarding Figure 3, the order of mean pupil diameters clearly shows the degree of pupil restrictions for stimuli. The order for patient with diseased eyes may be influenced by the disease. Table 2 shows mean pupil diameters and STDs for four stimuli across healthy subjects, patients with normal eyes, and patients with diseased eyes. The order of means is maintained across three types of eyes, and it does not seem easy to classify eye conditions using these statistics.

**Table 2:** **Mean pupil diameters for the stimuli**

	Healthy subject		Patient normal		Patient diseased	
	**Mean**	**STD**	**Mean**	**STD**	**Mean**	**STD**
r10	4.82	0.86	3.98	0.68	4.50	0.74
r100	4.30	0.90	3.41	0.73	3.99	0.74
b10	3.74	0.85	3.23	0.61	3.96	0.78
b100	3.16	0.85	2.65	0.69	3.18	0.74

During all observations for pupil reactions, repeated measures were not taken and so the test-retest reliability of the paradigm has not been evaluated.

### 3.3 Fourier descriptors

The feature vectors for PLR waveforms were extracted using the discrete Fourier transform (DFT) procedure [16],[17]. As mentioned above, PLRs were sampled as discrete signals. Here, the length *N* of a discrete signal is defined as *x*(*n*), which is sampled at time *t* with spacing *Δ*. The signal *x*(*n*) can be noted as an Equation 1 using DFT [32].

1 $$\begin{array}{ll} x(n) & =a_{0}+\overset{N/2}{\underset{k=1}{\sum }}\left(a(k)\text{cos}\left(2\mathrm{\pi k}\frac{t(n)}{\mathrm{N\Delta}}\right)\right.\;\\ \;\left.+\;b(k)\text{sin}\left(2\mathrm{\pi k}\frac{t(n)}{\mathrm{N\Delta}}\right)\right)\;\\ a_{0} & =X(1)/N\;\\ a(k) & =2\;\text{real}(X(k+1))/N\;\\ b(k) & =2\;\text{imag}(X(k+1))/N\; \end{array}$$

This suggests that PLR waveforms can be represented using coefficients *a*_0_, *a*(*k*), and *b*(*k*) with periodical sine and cosine functions. To present the features of the waveforms of pupillograms, the magnitudes of the coefficients are preferred because coefficient *b*(*k*) is the imaginary part of a value. The magnitudes of coefficients, including *a*_0_, FD_*i*_(*i*=0,…,*N*/2−1) are used as Fourier descriptors (FD) in vector (2) as follows:

2 $$f=[\;{\text{FD}}_{0},{\text{FD}}_{1},\ldots ,{\text{FD}}_{N/2-1}]$$

In general, the components *F* *D*_0_ and *a*_0_ in the Equation 1 show the DC components of the signal. These DC components represent the amplitude; however, the waveform shape consists of frequency components. Also, the features are affected by individual factors, so that a standardized feature using a component, such as FD_1_ for example, is preferred in vector (3), which is converted from the above vector (2), as follows [17]:

3 $$f=\left[\frac{{\text{FD}}_{2}}{{\text{FD}}_{1}},\frac{{\text{FD}}_{3}}{{\text{FD}}_{1}},\ldots ,\frac{{\text{FD}}_{N/2-1}}{{\text{FD}}_{1}}\right]$$

### 3.4 Prediction procedure for AMD disease

**Figure 5:**
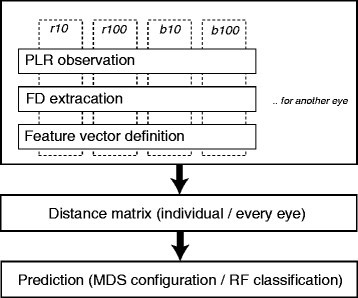
**Flowchart of signal processing and prediction.**

This paper proposes a procedure for detecting AMD patients and diseased eyes. The procedure is summarized in a flowchart in Figure 5. The first step is the definition of feature vectors of PLR waveforms for four stimuli using the FD technique mentioned above. Both eyes should be observed. The second step is to measure the (dis)similarity as distances between feature vectors of PLR waveforms for individuals for each eye. Finally, prediction in this paper is conducted using multidimensional scaling (MDS) and Random Forest techniques. The process of analysis is represented as follows.

Processing and prediction can be conducted for a few subjects without repeated measurements. Of course, the validity may not be sufficient, but it can be improved step by step when data from additional subjects is gathered. In this way, all of the techniques employed in this procedure are simple.

## 4Feature descriptions

### 4.1 Feature definition

The features of observed PLRs are presented using the procedure specified in the ‘Experimental procedure’ section. The actual calculations were conducted using MATLAB (Mathworks, Inc., Natick, MA, USA). To extract features of pupil constriction in transient and sustained pupil reactions, pupil responses mainly during the first 10 s are analyzed because they are melanopsin-based reactions. Every individual observation of the pupillogram for a stimulus was set to a signal *x*(*n*) in Equation 1, then FD vectors were extracted such as in Equation 2.

**Figure 6:**
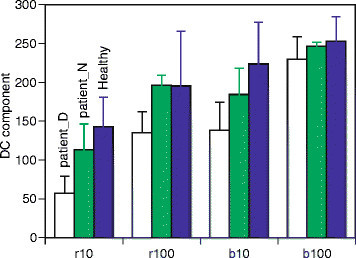
**Amplitudes of waveforms.**

First, FD_0_ were extracted in order to compare waveform amplitudes which were extracted from the transform. The amplitudes for PLR waveforms of all responses were calculated. Here, the amplitudes were standardized using the maximum peaks of the waveforms, such as b100, which suppresses individual differences for example [12]. The means were summarized into three categories: patients with diseased eyes, patients with normal eyes and healthy eyes. The results are shown in Figure 6. The error bars indicate standard errors. According to previous studies and Figure 1, the amplitude increases from the r10 condition to the b100 condition. The mean amplitude of healthy eyes responds to the order. However, these orders are influenced in patients with one normal and one diseased eye. Regarding Figures 1 and 2, pupil diameters at stimulus onset as baselines are different. The mean diameter for patients is 4.87 mm, and the mean for healthy subjects is 6.46 mm while there is a significant difference between them (*t*(10)=4.88,*p*<0.05). The factor of this difference is not determined, however. There is no significant difference in mean pupil diameters between patients with diseased and normal eyes (*t*(10)=0.60,*p*=0.56). The above result shows that the amplitudes present differences which are independent of baseline pupil diameters, since the differences change with the stimulus conditions in Figure 6.

The waveform shape can be noted as a vector in the Fourier descriptors section. A number of components of the feature vector represent the characteristics of most signals at only the low-order values of four or five FDs [16]. Therefore, four dimensions have been employed as feature vectors (*f*) in this paper. Also, FD_0_ was too large in this observation, so FD_1_ was used for the standardization. As an example, Fourier descriptors of r10 for the left eye of the healthy subject in Figure 1 are given as Equation 2:

4 $$f_{r10\text{\_}L}=[\;501.0,83.6,50.6,28.2,23.3,30.0,\ldots \;]$$

This vector is converted using Equation 3:

5 $$f_{r10\text{\_}L}=\left[\frac{50.6}{83.6},\frac{28.2}{83.6},\frac{23.3}{83.6},\frac{30.0}{83.6}\right]$$

Therefore, the feature vectors are noted as follows:

6 $$\begin{array}{ll} f_{r10\text{\_}L} & =[\;0.61,0.34,0.28,0.36]\;\\ f_{r100\text{\_}L} & =[\;0.68,0.52,0.40,0.36]\;\\ f_{b10\text{\_}L} & =[\;0.68,0.47,0.48,0.33]\;\\ f_{b100\text{\_}L} & =[\;0.64,0.43,0.33,0.30]\; \end{array}$$

### 4.2 Similarity/dissimilarity

As both Figures 1 and 2 show, the waveforms of PLRs in response to stimuli are different between subjects. To compare the shapes of waveforms quantitatively, the metrics of similarity and dissimilarity should be defined using waveform feature vectors which are noted above. This is a very popular approach for pattern recognition, such as categorization and discrimination of waveforms [33]. Here, the Euclidean distance (or Minkowski’s power metric) can be defined as the Euclidean norm between two feature vectors. This is the dissimilarity metric. The distances between stimuli conditions mentioned above for a set of PLRs of an eye (left eye) are summarized in the following matrix (Ed_*L*) as a triangular matrix.


7


These components indicate dissimilarity between the two waveform shapes when their amplitude factors are excluded.

## 5Comparing PLRs between eyes

### 5.1 Features of two eyes

There are many cases where one of the eyes is diseased and the other is not. As Table 1 shows visual acuities of both diseased (D) and normal (N) eyes, where all patients have a level of visual acuity in one eye that is comparable to healthy people and a diseased eye with poor acuity. Regarding the progress of AMD, the disease seldom progresses simultaneously in both eyes. This means that there is a difference in retinal condition between the left and right eyes of most patients with AMD. Since the retinal condition is different, the PLR waveforms are also different, as shown in Figures 1 and 2 while both healthy eyes respond similarly in Figure 1. This phenomenon suggests that quantitative differences in PLRs between two eyes may provide some information regarding symptoms of AMD. Therefore, a procedure of feature extraction from PLRs and the creation of a distance matrix in the above section can be applied to other cases with two eyes.

Here is a set of feature vectors of PLR waveform shapes of a patient:

8 $$\begin{array}{ll} f_{r10\text{\_}D} & =[\;10.54,0.22,0.21,0.17]\;\\ f_{r100\text{\_}D} & =[\;0.62,0.38,0.30,0.24]\;\\ f_{b10\text{\_}D} & =[\;0.59,0.37,0.25,0.20]\;\\ f_{b100\text{\_}D} & =[\;0.69,0.49,0.37,0.30]\;\\ f_{r10\text{\_}N} & =[\;0.65,0.23,0.23,0.14]\;\\ f_{r100\text{\_}N} & =[\;0.55,0.42,0.19,0.08]\;\\ f_{b10\text{\_}N} & =[\;0.23,0.56,0.29,0.26]\;\\ f_{b100\text{\_}N} & =[\;0.72,0.48,0.36,0.28]\; \end{array}$$

In this notation, *D* means diseased eye and *N* means normal eye for each experimental stimulus condition, such as r10.

Regarding the procedure and feature vectors, a distance matrix across two eyes for one subject can be created as follows:


9


To maintain the presentation of distance matrix for two eyes, both the left and right eyes (L/R) are allotted instead of diseased and normal eyes (D/N) for healthy subjects since they have two healthy eyes.

The distances between PLR waveforms for subjects with normal eyes are relatively shorter than the distances between diseased eyes and distances between one normal and one diseased eye. A matrix can be created for every subject based on this procedure.

According to the matrix of one patient, the distances representing the dissimilarity between the conditions for diseased eyes are longer than the distances for one normal eye and one diseased eye and the distances for two normal eyes. The distances for subjects with two healthy normal eyes are the shortest. Distance matrices were created for all subjects.

### 5.2 Configurations using MDS

To create an overall structure of the relationship between PLR waveforms, the MDS method [34] was applied to the distance matrix [35].

The basic approach of MDS is as follows:There are *n* samples which have feature vectors of waveforms, the distance can be defined using Euclidean distance such as *o*_*ij*_ between samples *i* and *j*. The diagonal distance components are 0. Here, a *n* by *A* matrix *X* of the waveform coordinates is introduced, another distance *d*_*ij*_ can be defined as the following equation:

10 $$d_{\text{ij}}={\left\{\overset{A}{\underset{a=1}{\sum }}{\left(x_{\text{ia}}-x_{\text{ja}}\right)}^{2}\right\}}^{1/2}$$

Additionally, the monotonic transformation of the data *g* is introduced, and the following equation is minimized:

11 $$\phi (g,\text{X})=\underset{j<i}{\sum }{\left(g(o_{\text{ij}})-d_{\text{ij}}\right)}^{2}$$

As a result, MDS produces a low-dimensional projection of the data which can present the paired distances between data points. As the low-dimensional projection provides a way to configure the data, the illustration can be used to visually understand the relationship between the data.

**Figure 7:**
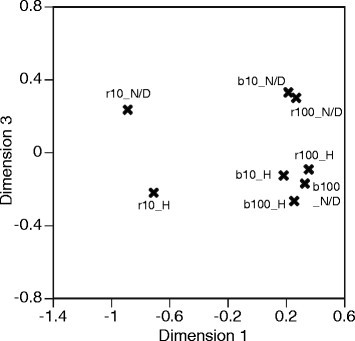
**Light pulse condition configurations using three-dimensional scales.**

The individual difference MDS procedure has been introduced to extend conventional MDS analysis to multiple distance matrices using the features of every subject [36],[37]. The actual calculation was conducted using R. During MDS analysis, the number of dimensions was set to three. Again, the dimensions are defined in order to present a low-dimensional projection regarding MDS calculations. Therefore, the dimension may present the features of the spatial layout of the data. The dimensional values are summarized in Table 3. The contributions of both dimension 1 and dimension 2 to the classification have the same tendency as in the case of a two-dimensional analysis. Following this, dimensions 1 and 3 were then compared. The stimulus is configured in a two-dimensional space which was created using MDS analysis, as shown in Figure 7. The horizontal axis shows dimension 1, and the vertical axis shows dimension 3. According to the distance matrix, one of the waveforms of a stimulus includes a diseased and a normal eye (D/N), the other includes eyes which are both healthy (H). The conditions are indicated in Figure 7 as ‘D/N’ for the former and ‘H’ for the latter. All normal conditions are gathered in one cluster except ‘r10H’, and only ‘b100-D/N’ belongs to a cluster which consists of normal responses. This suggests that for normal responses, stimuli conditions are configured outside of the cluster when the conditions include abnormal responses. This means that the possibility of detecting abnormal responses exists. According to the configuration, it is interesting that the r10 condition always shows a different tendency. This stimuli condition may provide significant information about the abnormal responses of diseased eyes.

**Table 3:** **Three-dimensional information of MDS for stimuli**

	D/N			H		
	dim1	dim2	dim3	dim1	dim2	dim3
r10	-.89	0.16	0.24	-.71	0.17	-.22
r100	0.27	0.26	0.30	0.35	0.37	-.09
b10	0.21	-.38	0.33	0.18	-.44	-.13
b100	0.33	-.05	-.17	0.25	-.08	-.26

D/N, diseased and normal eyes of AMD patients; H, healthy eyes.

**Figure 8:**
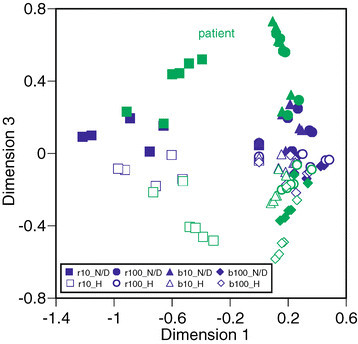
**The conditions for subjects configured using three-dimensional scales.**

**Figure 9:**
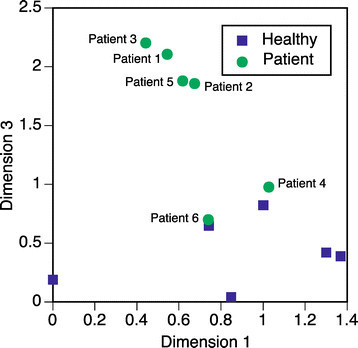
**The subjects’ configurations using three-dimensional scales.**

All results for the eight conditions of the six healthy subjects and six patients are mapped in Figure 8. The stimuli conditions produce clusters in response to the configurations of stimuli, as shown in Figure 8 where all subjects’ data is mapped in a similar style. However, the plots for the patient subjects are positioned in a different area. In particular, the values for patient subjects deviate from the norm in dimension 3. Even the plots of normal eyes of patient subjects are more widely distributed. The features of most patients show a different tendency. All subjects can be configured using their own individual two-dimensional information, as shown in Figure 9. In this figure, the value of dimension 3 clearly indicates the differences between healthy subjects and patient subjects. The healthy subjects produce a cluster in the lower area, and the patient subjects’ plots are distributed around the cluster. If the borders between the classes could be defined mathematically, classification according to health condition may also be possible. The meaning of dimension 3 cannot be defined mathematically in response to the nature of MDS analysis, however. The dimension is created as a deviation of distances each of the targeted data. A detailed analysis of this will be a subject of our further study.

**Figure 10:**
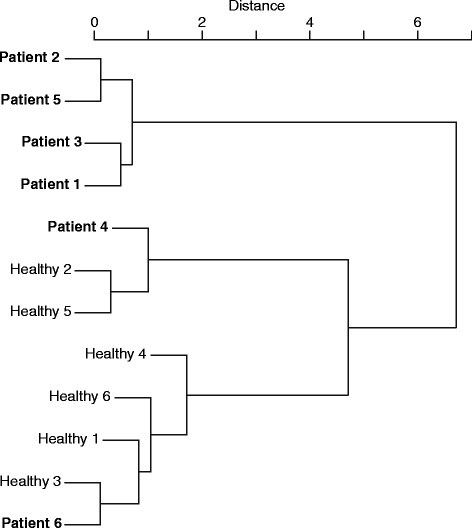
**Results of clustering of all subjects.**

Since MDS analysis also provides a three-dimensional feature for every subject and patient, grouping of participants is possible using cluster analysis and data of the feature set. The dendrogram as a result of cluster analysis is summarized in Figure 10. The horizontal axis shows averaged distance between subjects. The clustering process responds to the distribution of subjects in Figure 7. Most patients (the predominant type) are classified apart from other subjects.

This procedure can illustrate some of the differences between healthy subjects and patient subjects, and the clustering results show the tendency toward deviations. Therefore, the subject categories (healthy or patient) are ambiguous. Regarding the specific purpose of this work, more quantitative classification may be required as a result.

In the next section, a possible procedure for this will be introduced and discussed.

## 6Classification using a combination of amplitudes and features of waveform shapes of pupillograms

### 6.1 Feature combinations

In the previous section, the features of waveform shapes were extracted as amplitudes and Fourier descriptors. There were significant differences in the mean amplitudes between the eye conditions when the features of waveform shapes were used to separate the subjects. Though they can indicate some of the conditions affecting the eyes, they are not used collaboratively. Since they are recognized as different scales of features, some consideration is required in order to combine them. Also, a more robust classification technique should be considered.

To include FD_0_ components with features, the FD_0_ values were standardized using the means for each eye. In the case of the left eye of the healthy subject in Figure 1, the FD_0_^′^ of the standardized FD_0_ uses a mean of components. Both are noted as follows:

12 $$\;\begin{array}{cc} {{\text{FD}}_{0:L}}^{\prime } & =[\;{{\text{FD}}_{0:r10\text{\_}L}}^{\prime },{{\text{FD}}_{0:r100\text{\_}L}}^{\prime },{{\text{FD}}_{0:b10\text{\_}L}}^{\prime },{{\text{FD}}_{0:b100\text{\_}L}}^{\prime }]\\ \;[\;{{\text{FD}}_{0:r10\text{\_}L}}^{\prime },{{\text{FD}}_{0:r100\text{\_}L}}^{\prime },{{\text{FD}}_{0:b10\text{\_}L}}^{\prime },{{\text{FD}}_{0:b100\text{\_}L}}^{\prime },]\\ =[\;0.65,0.95,1.16,1.25] \end{array}$$

13 $$\begin{array}{ll} {{\text{FD}}_{0:L}}^{\prime } & =\frac{{\text{FD}}_{0:L}}{\frac{1}{n}|{\text{FD}}_{0:L}|}\;\\ {{\text{FD}}_{0:L}}^{\prime } & =[\;0.65,0.95,1.16,1.25]\; \end{array}$$

Combined vector *f*^′^ can be noted as a modified feature vector using weight coefficient *w*.

14 $$f^{\prime }=\left[\frac{{\text{FD}}_{2}}{{\text{FD}}_{1}},\frac{{\text{FD}}_{3}}{{\text{FD}}_{1}},\ldots ,\frac{{\text{FD}}_{5}}{{\text{FD}}_{1}},w{{\text{FD}}_{0}}^{\prime }\right]$$

Here, *w* is a coefficient used as a weighted value to create a balance between the features of waveform shapes and standardized FD_0_ (FD_0_^′^). To optimize the coefficient *w*, the performance of this classification is evaluated in response to variations of *w* in the section which follows.

### 6.2 Classification procedure

In this paper, the number of subjects is small, and the number of trials for taking measurements is limited because the experimental stimuli influences the response. The Random Forest method [38], which uses an ensemble learning procedure to analyze the classification of a small sample, is used frequently. Also, the Random Forest method can show contributions of features and can be used to conduct cross validation calculations. Since the structure of the feature data set is not clear, the results of the Random Forest method may provide useful information to improve the detection procedure. The statistical package R and the ‘RandomForest’ package [39],[40] were used for this analysis.

The number of decision trees was set at 500 as a default value, and the sample size was set at 6. The total number of samples was 24 (2×(6+6 subjects)). One third of the samples were assigned as test data, and the rest of data was assigned as OOB (Out of Bag) training data. The selection of the data set was initially random. This selection was performed 10 times, in order to calculate the generalized performance of the data throughout all conditions. To optimize the coefficient *w*, performance was evaluated according to the value of *w*, as follows:

15 w=[0,0.3,0.5,1.0,1.5,2.0,3.0,∞]

Here, *∞* means a case using only FD_0_^′^.

Classification of the modified set of features into two classes: healthy and normal (HN) and AMD (D), or into three classes: fealthy (H), AMD patient (D), and patient normal age-affected eyes (N) was conducted using the RF technique.

According to the preliminary analysis, the performance was low when the feature set of Equation 1 was used. Next, Euclidean distances between the four conditions were analyzed in the same way as in the previous assessment [41].

Here, a Euclidean distance matrix is shown as Ed_Healthy *L*_ for the example of the healthy subject’s left eye, and the distance feature is noted as FE. The FE vector consists of distance components without zero distance such as diagonal components in Ed matrix.


16

17 $$\begin{array}{l} \text{FE}=\;[\;r10-r100,r10-b10,r10-b100,r100\;\\ \;\;-b10,r100-b100,b10-b100]\;\\ {\text{FE}}_{\text{Healthy}\;L}=\;[\;0.37,0.57,0.62,0.24,0.33,0.18]\; \end{array}$$

### 6.3 Two-class performance

**Figure 11:**
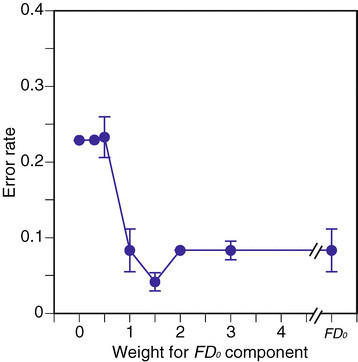
**Two-class classification error rate across** ***w*** **values.**

For two-class classification of PLRs of healthy and normal (HN) and AMD-affected diseased (D) eyes, 10 times calculations were conducted using *w* values. Mean error rates of classifications are summarized in Figure 11. The horizontal axis shows weight *w*, and the vertical axis shows the error rate. The error bar in the figure shows the standard deviation (STD). The minimum error rate appears at *w*=1.5. In the case of *w*=0.5 or only FD_0_^′^, the error rates are high.

The estimation performance for *w*=1.5 is summarized as a contingency table in Table 4. The table shows that healthy and normal eyes (HN) can almost always be correctly classified, but the error rate of classification for AMD-affected diseased eyes (D) is low, at 17%.

**Table 4:** **Contingency table for two classes**

	D	HN	Err.
AMD (D, *n* = 6)	5	1	0.17
Healthy and normal (HN, *n* = 18)	0	18	0.00

*w* for FD_0_’ = 1.5.

### 6.4 Three-class performance

**Figure 12:**
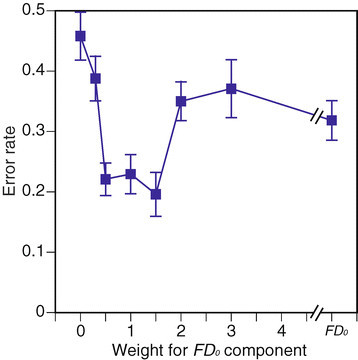
**Three-class classification error rate across** ***w*** **values.**

For three-class classification of PLRs of healthy (H), AMD-affected disease (D), and age-affected normal (N) eyes, 10 times calculations were conducted using *w* values. Mean error rates across *w* values are summarized in Figure 12 using the same format as in Figure 11. Error bars in the figure show the STDs of all 10 results. The minimum error rate also appears when *w*=1.5, while the rate changes with the *w* values.

The results for three classifications are summarized as a contingency table in Table 5, as mean rates for all 10 results. According to the results, healthy eyes are almost always correctly classified, while the error rate for AMD-affected diseased eyes is once again 17%. The performance for age-affected normal eyes is not good, as the error rate is over 50%. The age-affected normal eyes class may include eyes which respond in the same manner as healthy eyes or eyes which affected by AMD. Therefore, further observation of the condition of the subject’s eyes may be required.

**Table 5:** **Contingency table for three classes**

	D	N	H	Err.
AMD (D, *n* = 6)	5	0	1	0.17
Normal (N, *n* = 6)	1	3	2	0.50
Healthy (H, *n* = 12)	0	0.7	11.3	0.06

*w* for FD_0_’ = 1.5.

**Figure 13:**
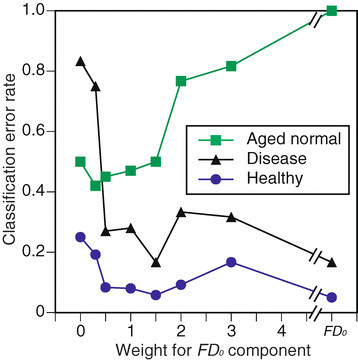
**Error rates for classification of each class.**

The error rates for the three-class classifications are summarized in Figure 13. The rates of both healthy and AMD-affected eyes are almost always small, and the minimum rate appears at *w*=1.5. The rates for age-affected eyes are relatively higher than the ones for the other two classes.

### 6.5 Features contributing to estimations

According to the classification results in the previous section, pupils can be classified accurately using features of PLR waveforms, except for the performance of age-affected normal eyes.

**Figure 14:**
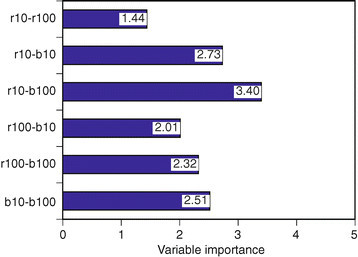
**Comparison of contribution values across features (** ***w*** **= 1.5).**

The next question was which components of features make it possible to classify PLRs. After that, the contributions of features were evaluated using the Random Forest tool. The degrees of contribution of each feature are summarized in Figure 14. The figure suggests that Euclidean distances are dissimilar between PLRs for blue light and PLRs for red light at low intensities such as *r* 10 to *b* 100 or *r* 10 to *b* 10 and denote the level of performance. Also, color differences at high intensities in PLRs such as *r* 100 to *b* 100 or *b* 10 to *b* 100 present the ability to distinguish the condition of the pupil.

These results coincide with the visual differences in PLRs between healthy and AMD-affected patients.

### 6.6 Clustered PLRs

**Figure 15:**
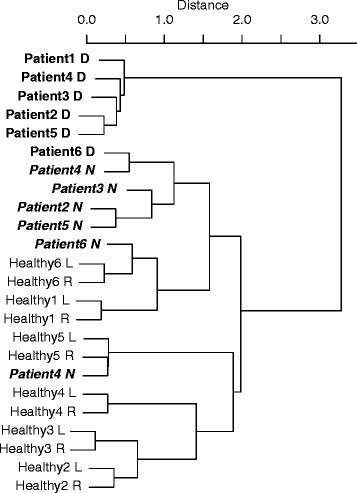
**Results of clustering of PLRs of all subjects (** ***w*** **= 1.5).**

The similarities of the features of waveform shapes in PLRs were also identified using the Random Forest procedure, and cluster analysis of these distances was then conducted using the Ward method. Figure 15 shows the resulting dendrogram of the clusters of PLR features with *w*=1.5 as the optimized value. In this figure, all subjects and eyes are indicated as healthy/patient and left/right or normal/diseased.

As the figure shows, the upper cluster displays a group of healthy subjects except for one patient with age-affected eyes. A sub-cluster consists of both eyes of most subjects. The lower clusters consist of patients with age-affected eyes in the upper part and patients with AMD-affected eyes in the lower part. Some healthy subjects and some patients with AMD-affected eyes have been incorrectly classified into these groups. These occurrences have been explained in the above section where performance was classified and evaluated.

To improve the performance of the test, responses should be ophthalmologically diagnosed. For the classification procedure, many different data mining tools have been developed, so a more effective procedure should be devised. The characteristics of features of pupillary response waveform shapes of pupillograms which result from using the Random Forest method will be a subject of our further study.

## 7Conclusions

This paper proposes a possible procedure for detecting AMD-affected eyes and age-affected eyes using features of PLR waveforms. The Fourier descriptor technique was applied to extract the features of PLR waveform shapes of pupillograms and their amplitudes.

To detect affected patients using the extracted features, MDS and clustering techniques were applied to emphasize certain stimuli and subject differences, since there were restrictions on gathering patient data. The performance is shown as a qualitative tendency.

To quantitatively evaluate detection performance, an appropriate classifier such as the Random Forest method was introduced. For this classification, a combination of features was used in order to obtain detailed features of waveform shapes of pupillograms. This was a balanced combination of the two features, and was controlled using weighted values. As a result of classification analysis using the Random Forest method, the performance of the three categories (patients, aged, and healthy subjects) was compared after the weighted values were optimized using variations of the classification error rate.

The results show that the error rates for healthy pupils and AMD-affected pupils were low when the value of the coefficient for a combination of PLR amplitudes and feature of waveforms was optimized as 1.5. However, the error rates for patients with age-affected eyes was not low.

The pupils of patients with age-affected eyes were influenced by various factors, so it may not be easy to classify healthy and AMD-affected patients in some cases. Additional feature processing, such as processing of the pupil diameter [42],[43] and having a sufficient number of patients for statistical analysis [11] may be required.

Also, the evaluation of additional diagnostic procedures will be a subject of our further study in the future.

## Authors’ original submitted files for images

Below are the links to the authors’ original submitted files for images. Authors’ original file for figure 1 Authors’ original file for figure 2 Authors’ original file for figure 3 Authors’ original file for figure 4 Authors’ original file for figure 5 Authors’ original file for figure 6 Authors’ original file for figure 7 Authors’ original file for figure 8 Authors’ original file for figure 9 Authors’ original file for figure 10 Authors’ original file for figure 11 Authors’ original file for figure 12 Authors’ original file for figure 13 Authors’ original file for figure 14 Authors’ original file for figure 15 Authors’ original file for figure 16 Authors’ original file for figure 17 Authors’ original file for figure 18
